# Recurrent pneumothorax developing during chemotherapy in a patient with miliary tuberculosis

**DOI:** 10.4103/1817-1737.36555

**Published:** 2007

**Authors:** Prem Parkash Gupta, Dinesh Mehta, Dipti Agarwal, Trilok Chand

**Affiliations:** *Department of Respiratory Medicine, Postgraduate Institute of Medical Sciences, Rohtak, India*; * Department of Physiology, Postgraduate Institute of Medical Sciences, Rohtak, India

**Keywords:** Computed tomogram features, miliary tuberculosis, nonimmunocompromised patient, recurrent pneumothorax

## Abstract

Despite the fact that miliary tuberculosis is frequently seen, associated pneumothorax developing during antitubercular chemotherapy for miliary tuberculosis is rare. Pneumothorax is potentially life threatening in association with miliary tuberculosis; and its symptoms may be masked by those of miliary tuberculosis, leading to avoidable delay in the diagnosis of pneumothorax. Here we describe a 24-year-old female patient developing recurrent pneumothorax while on antitubercular chemotherapy for miliary tuberculosis.

Miliary tuberculosis refers to clinical disease resulting from the uncontrolled hematogenous dissemination of *Mycobacterium tuberculosis*. The term ‘miliary’ was coined in 1700 by John Jacobus Manget, who described the appearance of the involved lung - with its surface covered with firm small white nodules resembling millet seeds. Originally a pathologic and then a radiological description, the term ‘miliary TB’ is now used to denote all forms of progressive, widely disseminated hematogenous tuberculosis, even if the classical pathological or radiological findings are absent.[1] Although miliary tuberculosis is a frequently seen disease, concomitant pneumothorax is a rare entity.[2] Pneumothorax is potentially life threatening in association with miliary tuberculosis; and as a patient with miliary tuberculosis also has dyspnea, the diagnosis of pneumothorax may be delayed.[3,4]

## Case Report

A 24-year-old woman presented at our department with complaint of low-grade intermittent fever and dry cough for 2 months that were not responding to antibiotics. She had no history of diabetes, hypertension or any other significant disease. Her routine investigations were within normal range. Chest radiograph taken at OPD was suggestive of miliary opacities [Figure 1A]. She had a positive tuberculin test (an induration of 18 × 18 mm) with 1 TU of PPD RT 23. She was diagnosed to have miliary tuberculosis and was admitted in the ward to evaluate other systems and to observe the response to antitubercular chemotherapy. She was prescribed WHO category I antitubercular chemotherapy (2H_3_R_3_Z_3_E_3_/4H_3_R_3_) along with other supportive treatment. After a few days, the fever subsided and there was clinical improvement seen in the patient. She was being planned for discharge from the ward, when her breathlessness increased. She had decreased breath sound over left lung. The chest radiograph confirmed the presence of pneumothorax [Figure 1B]. An intercostal drain under water seal was placed. By 48 h, the pneumothorax was reduced, the affected lung expanded and there was no water column movement at ‘under water seal’ drain. After having a stable course for over 2 days, the intercostal drain was withdrawn and she was discharged with advice to continue her antitubercular treatment and to avoid exertion. She remained clinically stable for 10 days, when she developed pain in the chest and her dyspnea increased. Her chest radiograph confirmed the recurrence of pneumothorax. Patient was readmitted and managed with intercostal drain under water seal for pneumothorax. Pleurodesis was done with tetracycline after her lung expanded. She was evaluated by high-resolution computed tomographic study, which showed miliary opacities [Figure 2A] and the presence of blebs over left lung apex [Figure 2B]. Her antitubercular chemotherapy was continued for 6 months. She had a stable course, with no recurrence of the pneumothorax.

**Figure 1A:**
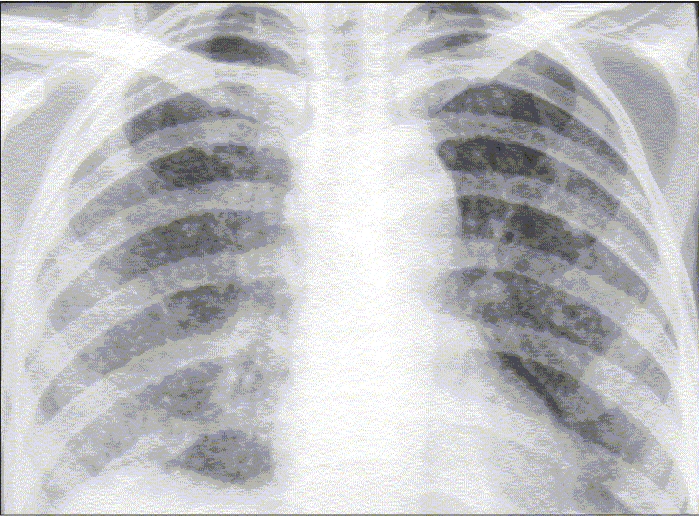
Chest radiograph frontal view showing miliary opacities over bilateral lung fields.

**Figure 1B:**
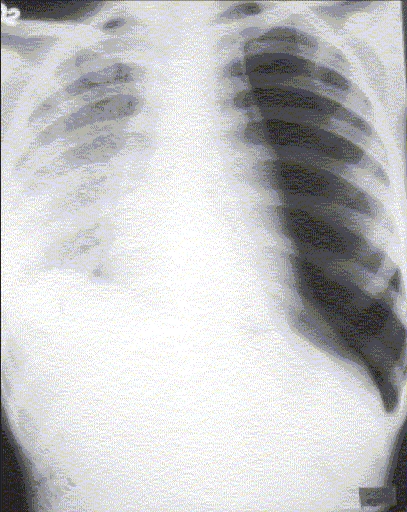
Chest radiograph frontal view showing left-sided pneumothorax.

**Figure 2:**
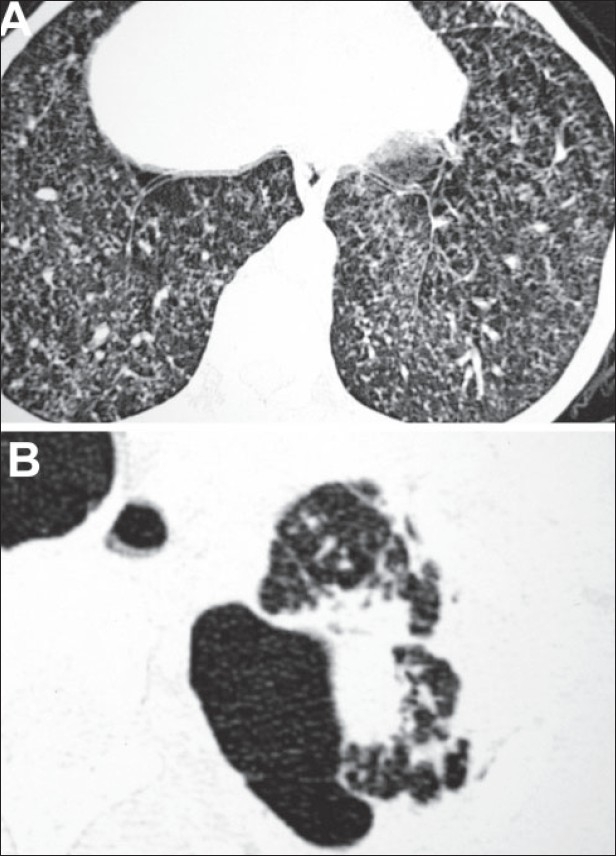
A. Computed tomogram revealing miliary opacities over bilateral lung fields. B. Computed tomogram showing multiple blebs over the left apex.

## Discussion

Almost a century ago, tuberculosis was regarded as the most significant etiological factor leading to pneumothorax.[5] Over a period of time, with the widespread use of imaging modalities and other diagnostic investigations and with a better understanding of the pathogenesis, it was realized that pneumothorax is but the cause of a fraction of pneumothoraces. At present pneumothorax is a frequent complication associated with cavitary tuberculosis, but it is rarely reported with miliary tuberculosis.[6]

The pathogenesis of pneumothorax in miliary tuberculosis remains elusive. Various possible mechanisms have been described. One probable mechanism is that the occurrence of pneumothorax along with subcutaneous emphysema is initiated due to excessive coughing associated with miliary tuberculosis, which results in a sudden increase in intra-alveolar pressure with concomitant airway narrowing. This leads the air into interstitial tissues of the lung and into the vascular adventitia of the hilum. The air in the mediastinum then leaks through the mediastinal pleura into the pleural space leading to pneumothorax. This mechanism may not explain the occurrence of isolated pneumothorax (without any pneumomediastinum) associated with miliary tuberculosis. Here the possible mechanism is formation of small areas of confluent subpleural miliary nodules. These subpleural miliary nodules undergo caseous necrosis with subsequent rupture into the pleural space. This will lead to air leak into pleural cavity, causing pneumothorax.[1,7,8] This has been the likely mechanism in the present case.

Although pneumothorax is rare, it is potentially life threatening in association with miliary tuberculosis. It is likely to be missed as the breathlessness and the dry coughing that are the cardinal features of pneumothorax are also seen in patients with miliary tuberculosis without any pneumothorax.[9] Frequently pneumothorax is not seen at the beginning of therapy but is seen during the course of treatment when it is least expected.[10] This warrants the treating physicians to be perceptive of the worsening of the clinical course of miliary tuberculosis when the patient presents with increasing dyspnea because of pneumothorax, which is a life-threatening emergency, maybe the underlying pathology,[2,10] and can be managed and treated, as in the present case, effectively.
